# Building the Science: Current Studies on the Impact of Arts Engagement on the Health of Older Adults

**DOI:** 10.1093/geroni/igaa057.2210

**Published:** 2020-12-16

**Authors:** Marie Bernard, Sunil Iyengar

**Affiliations:** 1 National Institutes of Health, Bethesda, Maryland, United States; 2 National Endowment for the Arts, Washington, District of Columbia, United States

## Abstract

Nearly a decade ago, a federal interagency task force on the arts and human development was launched as the result of a research summit held by the National Endowment for the Arts and the U.S. Department of Health and Human Services to investigate the arts’ relationships to health and well-being across the lifespan. Soon afterward, the National Institute on Aging partnered with the Arts Endowment and the National Academy of Sciences to identify research recommendations to benefit healthy aging and the treatment of neurodegenerative diseases in older-adult populations. While this session will revisit some of those findings, it also will share more recent advances in biomedical and behavioral research being conducted by a growing network of “Sound Health” researchers at the nexus of neuroscience, music, and health==with direct implications for the future of research on the arts and aging.

